# CORAZON: a web server for data normalization and unsupervised clustering based on expression profiles

**DOI:** 10.1186/s13104-020-05171-6

**Published:** 2020-07-14

**Authors:** Thaís A. R. Ramos, Vinicius Maracaja-Coutinho, J. Miguel Ortega, Thaís G. do Rêgo

**Affiliations:** 1 Programa de Pós-Graduação em Bioinformática, Bioinformatics Multidisciplinary Environment (BioME), Instituto Metrópole Digital, Universidade Federal do Rio Grande do Norte, Natal, Brazil; 2 Advanced Center for Chronic Diseases (ACCDiS), Facultad de Ciencias Químicas y Farmacéuticas, Universidad de Chile, Santiago, Chile; 3 Instituto Vandique, João Pessoa, Brazil; 4 Departamento de Bioquímica e Imunologia, Instituto de Ciências Biológicas, Universidade Federal de Minas Gerais, Belo Horizonte, Brazil; 5 Departamento de Informática, Centro de Informática, Universidade Federal da Paraíba, João Pessoa, Brazil

**Keywords:** Gene expression, Machine learning, Clustering, Normalization, Expression profiling, Transcriptome analysis, Non-coding RNAs, Web server

## Abstract

**Objective:**

Data normalization and clustering are mandatory steps in gene expression and downstream analyses, respectively. However, user-friendly implementations of these methodologies are available exclusively under expensive licensing agreements, or in stand-alone scripts developed, reflecting on a great obstacle for users with less computational skills.

**Results:**

We developed an online tool called CORAZON (Correlations Analyses Zipper Online), which implements three unsupervised learning methods to cluster gene expression datasets in a friendly environment. It allows the usage of eight gene expression normalization/transformation methodologies and the attribute’s influence. The normalizations requiring the gene length only could be performed to RNA-seq, meanwhile the others can be used with microarray and/or NanoString data. Clustering methodologies performances were evaluated through five models with accuracies between 92 and 100%. We applied our tool to obtain functional insights of non-coding RNAs (ncRNAs) based on Gene Ontology enrichment of clusters in a dataset generated by the ENCODE project. The clusters where the majority of transcripts are coding genes were enriched in Cellular, Metabolic, Transports, and Systems Development categories. Meanwhile, the ncRNAs were enriched in the Detection of Stimulus, Sensory Perception, Immunological System, and Digestion categories. CORAZON source-code is freely available at https://gitlab.com/integrativebioinformatics/corazon and the web-server can be accessed at http://corazon.integrativebioinformatics.me.

## Introduction

Gene expression is the process by which information encoded in a particular genomic region is transcribed in a functional gene product. These products can be coding or non-coding RNAs, i.e. transcripts that do not encode a protein but are functional important players in the cellular regulation in organisms from all domains of life [1–6]. Microarrays and RNA sequencing (RNA-seq) are large-scale technologies commonly used to measure transcript expression levels [7–12]. Both technologies generate a final expression matrix, containing the raw values for all biological samples in a study, which will be subsequently used in order to obtain the set of differentially expressed transcripts in studied samples and conditions.

The values of gene expression can be influenced by different variables (i.e. biological conditions, expression technology, sequencing library length, RNA quality), disproportionating the number of reads/hybridizations associated with particular samples, affecting the real expression values of studied samples. For a proper and reliable interpretation of quantitative gene expression measurements, a normalization is necessary to correct expression bias generated by these variables. Different data normalization approaches have been described so far. For instance, in many studies, a single housekeeping gene is used for normalization. However, no unequivocal single reference gene or non-coding RNA (with a proven invariable expression between cells and conditions) has been described yet [13]. As an alternative, the mean expression of multiple genes can be used for normalization [13, 14]. In RNA-seq, gene expression values are normally normalized by the size of the library.

The large quantity of biological data generated in large-scale genomics and transcriptomics projects thrived an intense demand to use computational techniques provided by artificial intelligence [15–18]. Unsupervised learning is the machine learning task of inferring a function to describe the hidden structure from unlabeled data. The inference of the function is performed with the analysis of gene expression, in which commonly, genes with the same expression patterns at the same time points and conditions can be participating on the same biological processes. Unsupervised methods transform the gene expression data on coordinates of a point in a given space and cluster them according to their similarities. The method uses the examples provided and tries to determine if some of them can be grouped in any way, forming clusters. Gene expression clustering has the goal to subdivide sets of expressed transcripts in such a way that those with similar expression patterns fall into the same cluster, while those with different expression patterns fall into different clusters [19]. It allows a deeper exploration of the data. For instance, transcripts co-expressed in a set of different experiments or conditions tend to be part of the same biological pathways and may possess similar gene ontology categories [20–25]. It is helpful in the functional assignation of transcripts without any functional annotation, as well as on the identification of co-regulated transcripts.

Packages available in R, Perl or Python libraries provide normalization and clustering methods that can be used for gene expression analysis. However, to use these tools it is necessary prior knowledge in these programming languages, reflecting in a great obstacle for users with less computational or bioinformatics backgrounds. Here, we introduce a tool called CORAZON (Correlation Analyses Zipper Online), a user-friendly web server, developed to facilitate expression data normalization and clustering in a streamlined way, and applied it to obtain functional insights of ncRNAs based on their expression patterns and gene ontology enrichment.

## Main text

### Materials and methods

#### CORAZON implementation and clustering methods validation using simulated data sets

CORAZON web server was developed with eight normalization/transformation methodologies (https://corazon.integrativebioinformatics.me/documentation.html): Trimmed Mean of M-values (TMM) [26], Median Ratio Normalization (MRN) [27], Fragments Per Kilobase Million (FPKM), Transcripts Per Million (TPM), Counts Per Million (CPM), base-2 log, instance normalization and normalization by the highest attribute value for each instance. The normalizations which demand the transcript size (e.g. FPKM and TPM), we assumed that the 2^nd^ column will have this value. Moreover, three unsupervised machine learning algorithms (Mean Shift, *K*-Means and Hierarchical) adopting Euclidean distance a measure of similarity, and a strategy to observe the attributes influence in the results were incorporated.

Normalizations, the clustering algorithms K-Means and Mean Shift and the web server application were implemented using Python. Hierarchical clustering was implemented using R. MySQL language was used to store and query the job results, as well as to perform the communication and interaction with the web page. The interface was developed using HTML, CSS, Bootstrap, and Javascript. CORAZON source code with a Docker platform is freely available at https://gitlab.com/integrativebioinformatics/corazon and the web server can be accessed at http://corazon.integrativebioinformatics.me.

Implemented algorithms had their performances evaluated through five models commonly used to validate clustering methodologies. Simulated models were built based on the work of [28, 29]. For each model, we generated 50 datasets and applied the three algorithms implemented.

#### Application using expression data of human coding and non-coding transcripts

We used our tool to study an RNA-seq dataset of 13 different tissues extracted from ENCODE [30]. Our goal was to obtain functional insights for ncRNAs, through the exploration of gene ontology functional categories of protein-coding genes co-expressed with ncRNAs. The expression matrix for all 13 tissues was extracted from [30]. Data were normalized using TPM and log_2_, and clustered using the three available algorithms.

### Results

#### CORAZON web server overview and usage

**Fig. 1:**
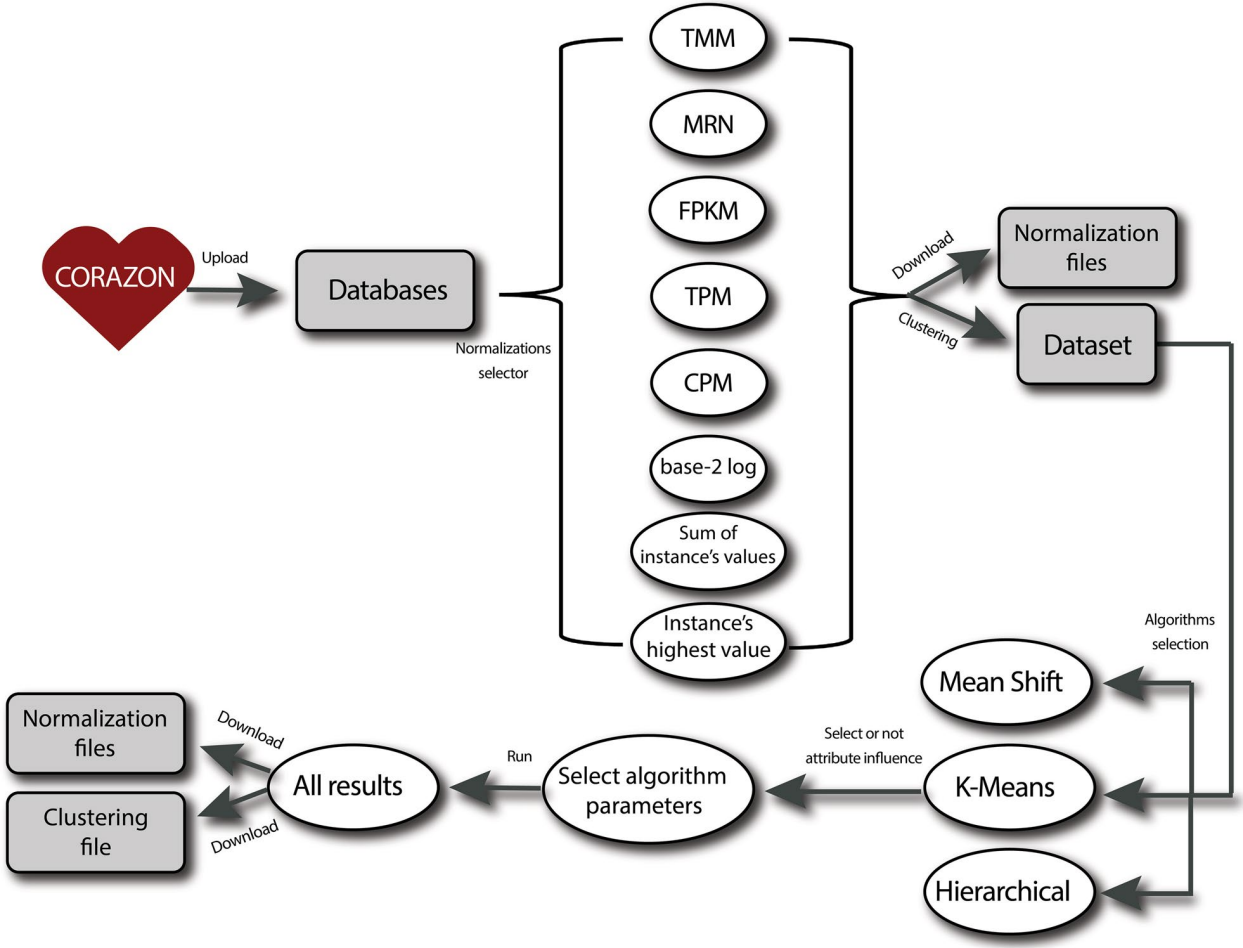
CORAZON whole workflow. Input and output files are shown in gray blocks; white circles represent the normalization methods, clustering algorithms and parameters selection

CORAZON is a streamlined web server that facilitates data normalization and uses machine learning to cluster transcripts according to their expression patterns. It receives as input an expression matrix, which can be used for different tasks, according to user preference. Briefly, the user can use the tool for only normalize their expression data, clustering the transcripts according to their expression patterns or both. Figure 1 shows the workflow of CORAZON tool.

#### Algorithms performance evaluation using simulated data

The implemented clustering algorithms had their performances evaluated through five models commonly used to validate clustering methodologies [28, 29]. The first model was the creation of 200 points in 10 dimensions; in the second we created 3 clusters in 2 dimensions; the third consists of generating 4 clusters in 3 dimensions; in the fourth we produced 4 clusters in 10 dimensions; and in the last model we had 2 elongated clusters in 3 dimensions. Thus, we generated 50 datasets and applied the three algorithms implemented in CORAZON web server. The algorithms presented accuracies ranging between 92 and 100%.

#### Functional insights of non-coding RNAs based on their expression patterns and gene ontology enrichment

**Fig. 2:**
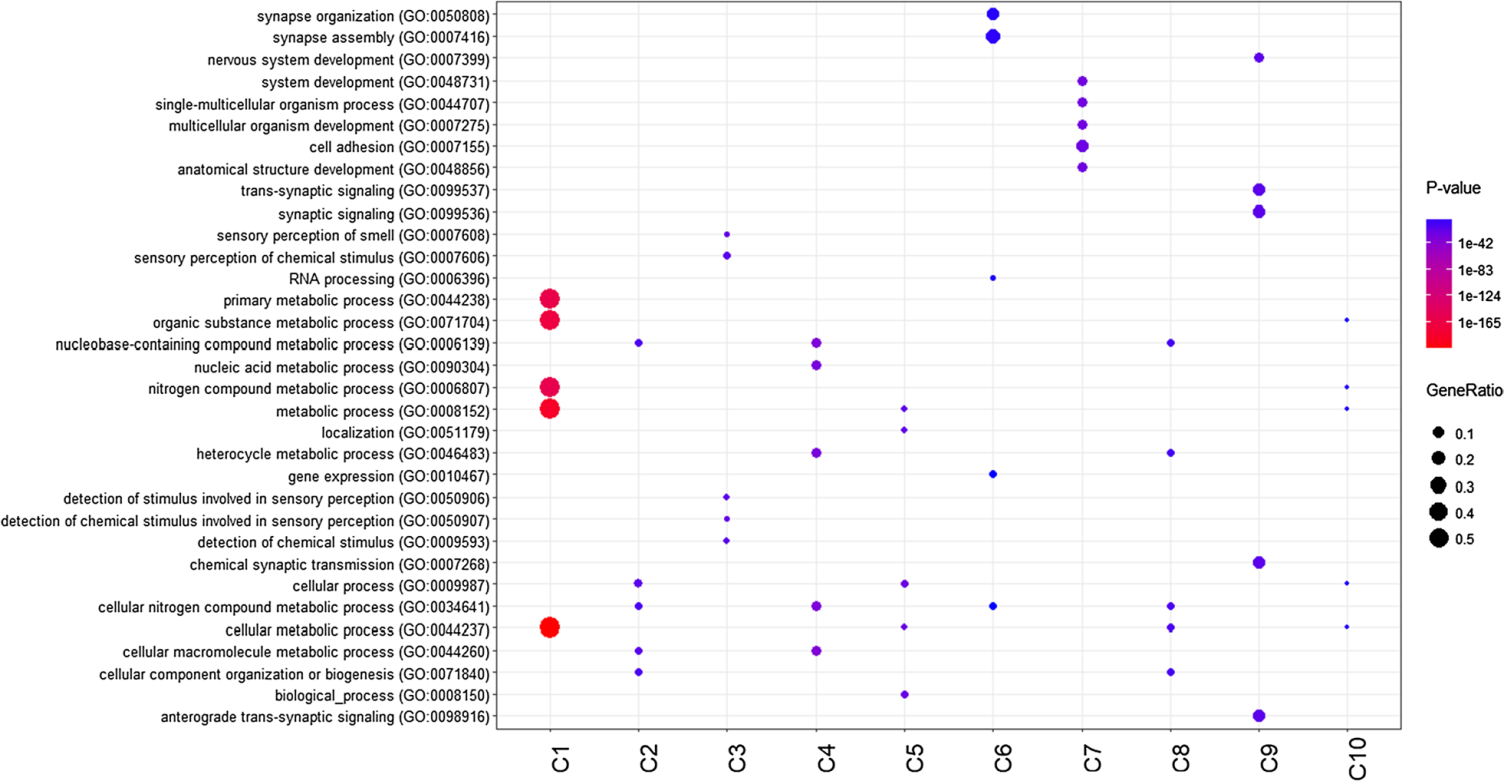
Enrichment analysis of Hierarchical clustering results. The x-axis represents the clusters found in this particular analysis, while the y-axis corresponds to the set of biological processes (GO terms) enriched in each cluster

We applied CORAZON to obtain functional information of ncRNAs based on the Gene Ontology enrichment of protein coding genes clustered together with ncRNAs, using a dataset composed of 13 RNA-seq assays from different human tissues generated by the ENCODE project. To select the best number of clusters for *K*-means and hierarchical algorithms, we used the Bayesian information criterion (BIC) [31], followed by the derivative of the discrete function and Silhouette [32]. In the hierarchical method, we tested 8 linkage criteria and adopted Ward’s method [33]. In total, we analyzed 41,283 transcripts (19,912 coding; 21,371 non-coding), which were clustered in 10 (*K*-means and hierarchical) and 13 (mean shift) clusters (Additional file 1: Table S1). The analysis using the three implemented algorithms identified sets of clusters represented mostly (more than 70%) by non-coding RNAs. Thus, GO enrichment analysis of the clusters composed in its majority by coding genes were usually enriched in cellular, metabolites, detection of stimulus, sensory perception, and systems development categories. The clusters composed in its majority by ncRNAs were enriched in coding genes associated with reproduction, development, immunological system, neurological system, localization, and digestion categories. An example of these results for hierarchical clustering can be found in Fig. 2. Results for *K*-means and mean shift can be found in Additional file 1: Figures S1 and S2, respectively.

**Fig. 3:**
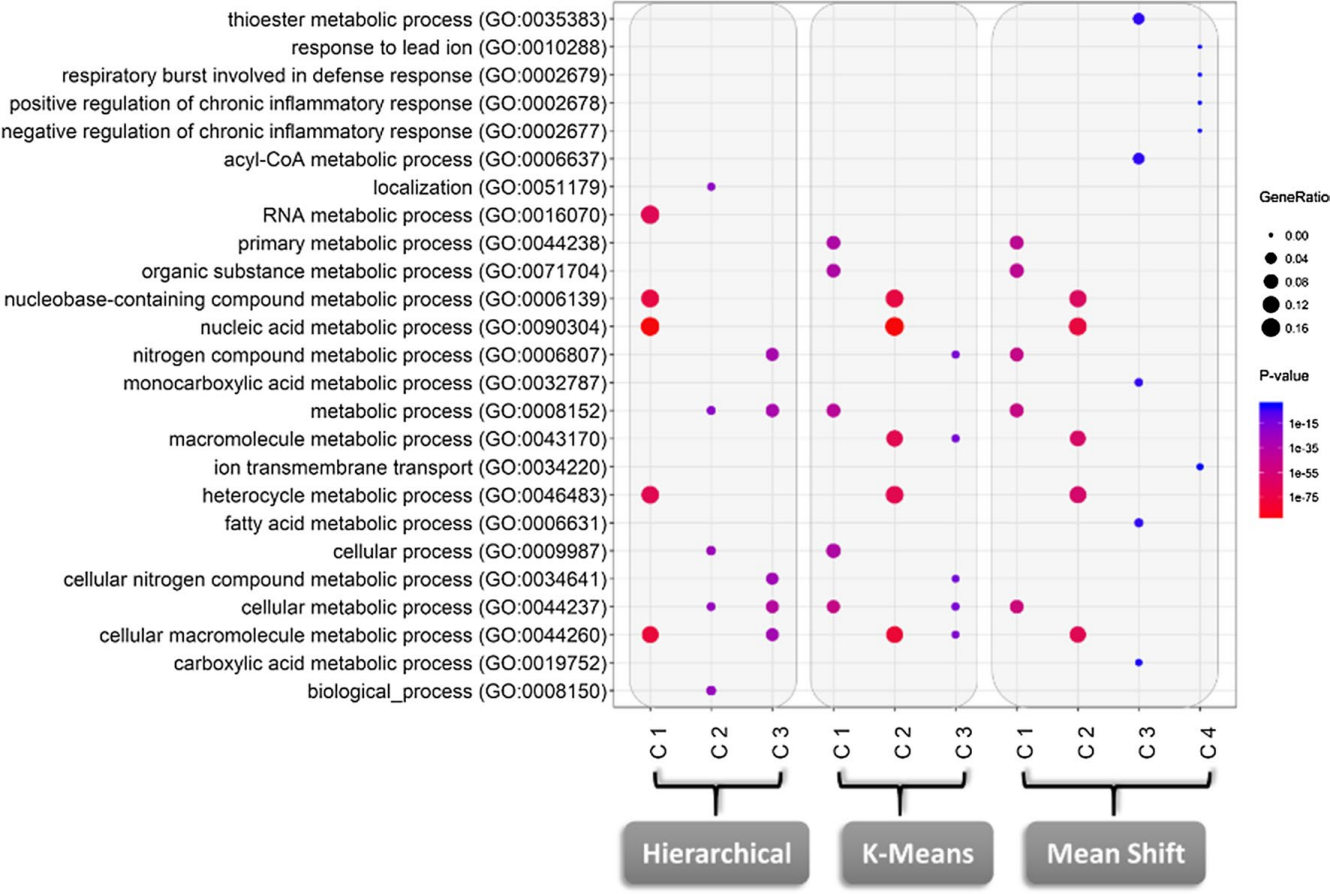
Enrichment of the ENCODE clusters generated by the three algorithms. The x-axis represents the clusters found in this particular analysis, while the y-axis corresponds to the set of biological processes (GO terms) enriched in each cluster

To gain further insights on the putative biological relevance of ncRNAs with correlated expression levels with coding genes, we used the three implemented algorithms to generate clusters of highly correlated transcripts (i.e. Spearman > 0.95). The correlation analysis revealed a set of 17,732 correlated transcripts (4829 coding genes and 12,903 non-coding RNAs). Hierarchical and *K*-means algorithms generated three clusters, meanwhile mean shift generated four (Additional file 1: Table S2). The algorithms generated two clusters composed mainly by non-coding RNAs (more than 50%). The gene ontology enrichment analysis revealed that these clusters were associated with coding genes related to different metabolic processes, localization and inflammatory and defense responses (Fig. 3).

### Discussion

CORAZON implemented normalization/transformation methodologies that can be used in RNA-seq, microarray and/or NanoString nCounter. It is worth to note that microarray and NanoString can only use the normalization methods that do not requires the transcript size. Those methodologies can normalize gene expression taking into account the different characteristics of the data (i.e. sequencing depth, transcript length, samples with disproportionate expression values). We successfully applied the tool to characterizing the expression patterns of coding and non-coding genes from 13 different tissues generated by the ENCODE project. Co-expressed transcripts are normally part of common biological pathways and functional GO categories, or they can be regulated by similar mechanisms [20–25]. Firstly, all 41,283 expressed coding (19,912) and non-coding (21,371) transcripts were clustered according to their expression values, using the three unsupervised clustering algorithms incorporated in CORAZON. This analysis revealed 10 clusters for hierarchical and *K*-means algorithms and 13 clusters for the mean shift algorithm. GO analysis revealed that most of the clusters generated by the three algorithms are enriched with similar biological process categories, associated with key general processes from the cell (i.e. metabolic processes, transport, systems development, detection of stimulus, RNA processing, sensory perception, immunological system, digestion, reproduction, synaptic signaling, neurological system and defense response). Thus, the similarity in the results (from hierarchical to partition methods) of the clusters enrichment analysis, strengthens the hypothesis that these transcripts actually have similar biological processes.

Furthermore, we observed that clusters enriched with coding genes (i.e. composed by more than 80% of coding genes) are related to GO terms associated with general metabolic processes, development, and cell adhesion. Clusters enriched with ncRNAs (i.e. more than 70% of non-coding genes) are related to coding genes associated with reproduction, immunological system, neurological system, localization, and digestion. Those results suggest that the set of ncRNAs clustered together with coding genes that are associated with the functional categories listed above could also be part of biological cellular processes directly linked to these mechanisms. The performance of ncRNAs in most of these processes have been widely studied, revealing its role in regulating proper cell functioning or disease (i.e. neurological disorders and cancers) [34–41]. For instance, [42] used the enrichment of functional GO annotations of coding genes located in the vicinity to ncRNAs, and noted that the two groups with the highest number of ncRNAs were associated with “synaptic transmission” (47 non-coding RNAs) and “generation of male gametes” (20 ncRNAs). This finding is consistent with previous studies and reinforce that ncRNAs are particularly active in the brain or during embryonic development.

Using CORAZON to cluster highly correlated transcripts (i.e. Spearman > 0.95), each algorithm generated two clusters represented in its majority by ncRNAs (more than 50%). Those clusters were associated with different metabolic processes, localization, inflammatory and defense responses. It was also observed that other clusters had specificities in cellular, metabolic, localization, transport and response processes. Finally, it was observed that clusters composed in its majority by coding genes (i.e. more than 82%) were related to metabolic processes. It was also observed that hierarchical cluster 1 (with 93.33% of coding genes) and *K*-means cluster 2 (with 93.69% of coding genes) were almost identical.

In summary, CORAZON simplifies gene expression normalization and unsupervised clustering. The results obtained in this study illustrate the potential of the tool and the possibilities of obtaining functional insights from clusters through the use of predictive associations between ncRNAs and the functional categories of clustered together coding genes. There are other methodologies for gene expression data normalization available in literature (e.g. quantile and RMA for microarrays; RLE for RNA-seq [43, 44]) that are not yet incorporate in our tool, but we intend to implement in the close future.

## Limitations

CORAZON architecture works with a process queue, resulting in a potential long-time waitlist for the user if we have hundreds of users at the same time. We are currently working on the parallelization of the tool to avoid this issue.

## Supplementary information

**Additional file 1.** Additional figures and tables.

## Data Availability

Some of the data analysed during this study were obtained from the article: Lin S, Lin Y, Nery JR, Urich MA, Breschi A, Davis CA, Dobin A, Zaleski C, Beer MA, Chapman WC, Gingeras TR, Ecker JR, Snyder MP: Comparison of the transcriptional landscapes between human and mouse tissues. Proceedings of the National Academy of Sciences of the United States of America. 2014, 48:17224-17229. 10.1073/pnas.1413624111

## Abbreviations

BIC: Bayesian Information Criterion
CORAZON: Correlations Analyses Zipper Online
CPM: Counts Per Million
CSS: Cascading Style Sheets
FPKM: Fragments Per Kilobase
HTML: Hypertext Markup Language
ID: Job Identifier Number
MRN: Median Ratio Normalization
mRNA: Messenger RNA
MySQL: My Structured Query Language
ncRNA: Non-coding RNA
RNA: Ribonucleic acid
RNA-Seq: RNA sequencing
TMM: Trimmed Mean of M-values
TPM: Transcripts Per Million
