# Supplementary material for: CORAZON: a web server for data normalization and unsupervised clustering based on expression profiles
Source: BMC Res Notes. 2020 Jul 14;13:338. doi: 10.1186/s13104-020-05171-6 (PMC7359491; doi:10.1186/s13104-020-05171-6)
Supplement: Supplementary file 1 — Additional file 1. Additional figures and tables. [file 13104_2020_5171_MOESM1_ESM.docx]

**Additional Material**

**Figure S1.** Enrichment analysis of *K*-Means clustering results. The x-axis represents the clusters found in this particular analysis, while the y-axis corresponds to the set of biological processes (GO terms) enriched in each cluster.

**
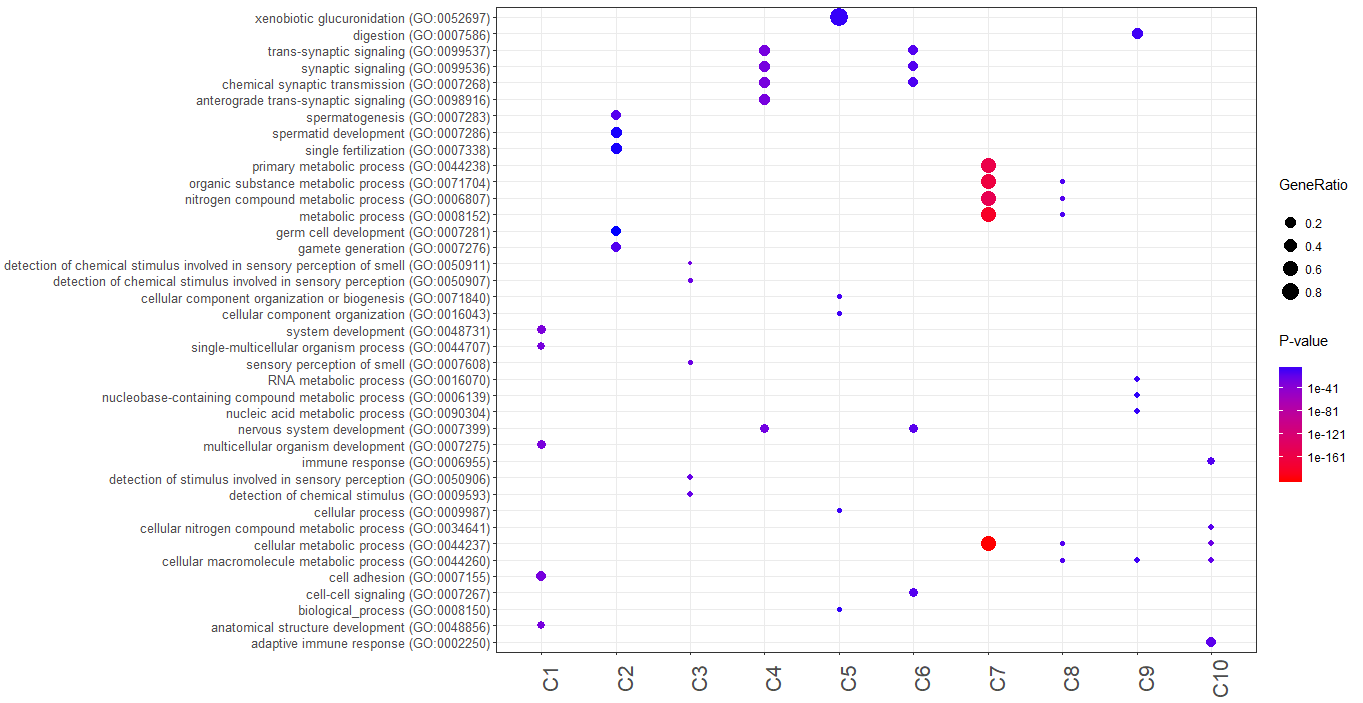
**

**Figure S2.** Enrichment analysis of Mean Shift clustering results. The x-axis represents the clusters found in this particular analysis, while the y-axis corresponds to the set of biological processes (GO terms) enriched in each cluster.


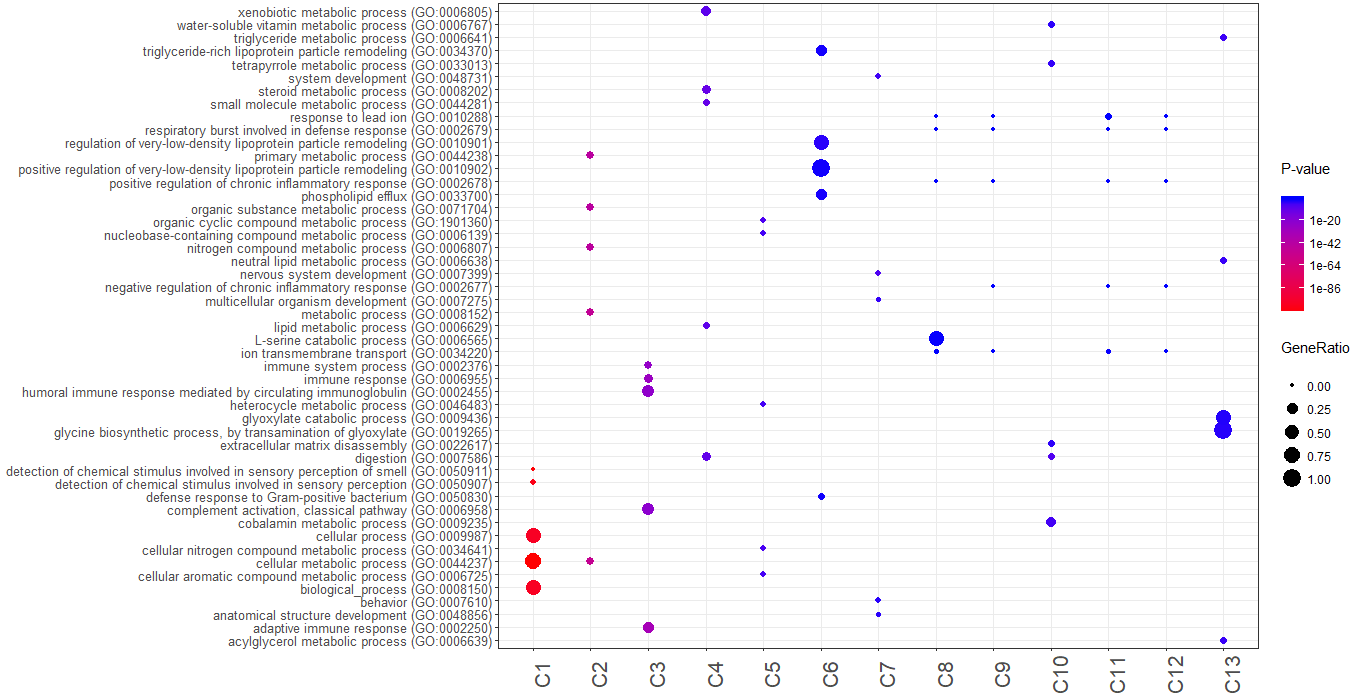


**Supplementary Table 1.** Results description generated by the three clustering algorithms. The first column refers to the clusters identifiers; the second and third columns represent the number of genes of each class (coding or non-coding) present in the given cluster; and the fourth and fifth columns show the percentage of genes of each class that compose each cluster.

| ***HIERARCHICAL*** | | | | |
| --- | --- | --- | --- | --- |
| *Cluster* | **Coding** | **Non-Coding** | **% Coding** | **% Non-Coding** |
| *1* | 8257 | 271 | 96.82 | 3.18 |
| *2* | 1198 | 3241 | 26.99 | 73.01 |
| *3* | 3383 | 1800 | 65.27 | 34.73 |
| *4* | 2730 | 3726 | 42.29 | 57.71 |
| *5* | 830 | 4819 | 14.69 | 85.31 |
| *6* | 777 | 567 | 57.81 | 42.19 |
| *7* | 908 | 202 | 81.80 | 18.20 |
| *8* | 1087 | 4944 | 18.02 | 81.98 |
| *9* | 640 | 1203 | 34.73 | 65.27 |
| *10* | 102 | 598 | 14.57 | 85.43 |
| ***K-MEANS*** | | | | |
| *Cluster* | **Coding** | **Non-Coding** | **% Coding** | **% Non-Coding** |
| *1* | 1318 | 477 | 73.43 | 26.57 |
| *2* | 1525 | 7267 | 17.35 | 82.65 |
| *3* | 3754 | 2190 | 63.16 | 36.84 |
| *4* | 996 | 1344 | 42.56 | 57.44 |
| *5* | 435 | 1723 | 20.16 | 79.84 |
| *6* | 1258 | 961 | 56.69 | 43.31 |
| *7* | 8344 | 286 | 96.69 | 3.31 |
| *8* | 626 | 3613 | 14.77 | 85.23 |
| *9* | 806 | 1700 | 32.16 | 67.84 |
| *10* | 850 | 1810 | 31.95 | 68.05 |
| ***MEAN SHIFT*** | | | | |
| *Cluster* | **Coding** | **Non-Coding** | **% Coding** | **% Non-Coding** |
| *1* | 13618 | 3883 | 77.81 | 22.19 |
| *2* | 2915 | 12863 | 18.48 | 81.52 |
| *3* | 1394 | 2232 | 38.44 | 61.56 |
| *4* | 401 | 641 | 38.48 | 61.52 |
| *5* | 666 | 771 | 46.35 | 53.65 |
| *6* | 119 | 475 | 20.03 | 79.97 |
| *7* | 179 | 353 | 33.65 | 66.35 |
| *8* | 102 | 113 | 47.44 | 52.56 |
| *9* | 46 | 109 | 29.68 | 70.32 |
| *10* | 24 | 27 | 47.06 | 52.94 |
| *11* | 30 | 88 | 25.42 | 74.58 |
| *12* | 34 | 119 | 22.22 | 77.78 |
| *13* | 29 | 52 | 35.80 | 64.20 |

**Supplementary Table 2.** Clusters composition in relation to coding and non-coding genes.

| ***HIERARCHICAL*** | | | | |
| --- | --- | --- | --- | --- |
| *Cluster* | **Coding** | **Non-Coding** | **% Coding** | **% Non-Coding** |
| *1* | 1623 | 116 | 93.33 | 6.67 |
| *2* | 830 | 4819 | 14.69 | 85.31 |
| *3* | 2376 | 7968 | 22.97 | 77.03 |
| ***K-MEANS*** | | | | |
| *Cluster* | **Coding** | **Non-Coding** | **% Coding** | **% Non-Coding** |
| *1* | 2500 | 8906 | 21.92 | 78.08 |
| *2* | 1618 | 109 | 93.69 | 6.31 |
| *3* | 711 | 3888 | 15.46 | 84.54 |
| ***MEAN SHIFT*** | | | | |
| *Cluster* | **Coding** | **Non-Coding** | **% Coding** | **% Non-Coding** |
| *1* | 3438 | 12500 | 22.57 | 78.43 |
| *2* | 1277 | 273 | 82.39 | 17.51 |
| *3* | 27 | 12 | 69.23 | 30.87 |
| *4* | 87 | 118 | 42.44 | 57.56 |
